# Earthworm symbiont *Verminephrobacter eiseniae* mediates natural transformation within host egg capsules using type IV pili

**DOI:** 10.3389/fmicb.2014.00546

**Published:** 2014-10-29

**Authors:** Seana K. Davidson, Glenn F. Dulla, Ruth A. Go, David A. Stahl, Nicolás Pinel

**Affiliations:** ^1^Department of Civil and Environmental Engineering, University of Washington, Seattle, WAUSA; ^2^Institute for Systems Biology, Seattle, WAUSA

**Keywords:** earthworm, *Verminephrobacter*, natural transformation, type IV pili, horizontal gene transfer, symbiosis

## Abstract

The dense microbial communities commonly associated with plants and animals should offer many opportunities for horizontal gene transfer through described mechanisms of DNA exchange including natural transformation (NT). However, studies of the significance of NT have focused primarily on pathogens. The study presented here demonstrates highly efficient DNA exchange by NT in a common symbiont of earthworms. The obligate bacterial symbiont *Verminephrobacter eiseniae* is a member of a microbial consortium of the earthworm *Eisenia fetida* that is transmitted into the egg capsules to colonize the embryonic worms. In the study presented here, by testing for transformants under different conditions in culture, we demonstrate that *V. eiseniae* can incorporate free DNA from the environment, that competency is regulated by environmental factors, and that it is sequence specific. Mutations in the type IV pili of *V. eiseniae* resulted in loss of DNA uptake, implicating the type IV pilus (TFP) apparatus in DNA uptake. Furthermore, injection of DNA carrying antibiotic-resistance genes into egg capsules resulted in transformants within the capsule, demonstrating the relevance of DNA uptake within the earthworm system. The ability to take up species-specific DNA from the environment may explain the maintenance of the relatively large, intact genome of this long-associated obligate symbiont, and provides a mechanism for acquisition of foreign genes within the earthworm system.

## INTRODUCTION

Within any microbial community there is potential for horizontal gene transfer (HGT) through several described mechanisms of DNA exchange. In addition to exchange facilitated by viruses and virus-like particles, DNA transfer among bacteria commonly occurs via mating pili (conjugation) and through uptake of free DNA from the environment by competent cells (natural competence/transformation; (Lorenz and Wackernagel, 1994). Natural competence, initially discovered in a mouse pathogen (Griffith, 1928), has been documented in both gram positive and gram negative bacteria, including pathogenic and free-living bacteria (Chen and Dubnau, 2004; Thomas and Nielsen, 2005; Johnsborg et al., 2007). Free DNA, shed from both live and dying cells, is estimated to range from 1 μg per gram of soil to 0.03–88 μg per litre of water, and is also found in biofilms as a structural component (Baur et al., 1996; Conover et al., 2011; Barnes et al., 2012), providing material for HGT throughout microbial communities. Well-studied naturally competent organisms include *Neisseria* species, *Streptococcus pneumonia*, *S. sanguis*, *Pseudomonos stutzeri*, *P. aeruginosa, Bacillus subtilis*, *Haemophilus influenza, Streptomyces* (Sisco and Smith, 1979; Stewart and Carlson, 1986; Elkins et al., 1991; Smith et al., 1995; Johnsborg et al., 2007), and *Acinetobacter baylyi* (Palmen et al., 1993; Vaneechoutte et al., 2006), many of which are associated with host organisms as pathogens. The ability to take up naked DNA is hypothesized to serve as a source for nutrition and material for DNA repair (Dubnau, 1999), but has also been demonstrated to enable adaptation to changing conditions by acquisition of new genes resulting in new capabilities (Johnsborg et al., 2007).

Among free-living microorganisms, the genetic fodder for innovation is provided by the microbial diversity in the local environment. However, microbial symbionts transferred strictly through vertical inheritance, and therefore lacking a free-living stage exposed to the open environment, have limited access to a gene pool outside their immediate population. Thus, the genomes of most obligate symbionts isolated within host cells are subject to rapid erosion through drift, bottlenecking, and irreversible loss of functional independence (Ochman and Moran, 2001; Moran et al., 2009; Lund et al., 2014). In contrast, mutualisms that include a free-living stage retain the opportunity for exchange with environmental populations. Although HGT among members of host-associated communities occurs (Ochman and Moran, 2001; Finan, 2002; Piel et al., 2004; Nandasena et al., 2007) the significance of exchange among bacteria leading to enhancement or disruption of beneficial interactions between microbial mutualists and their hosts has not been well-documented. Given the prevalence of natural transformation (NT) and DNA exchange among pathogens, it is surprising that NT has only recently been demonstrated in culture for one beneficial bacterial symbiont, *Vibrio fischeri* of the squid light organ (Pollack-Berti et al., 2010). Natural competence is induced in *V. fischeri*, and in *V. cholerae*, by chitin polymers, a natural component of the hosts of both bacteria (squid and shellfish). Chitin is common in sea water, suggesting uptake of DNA also occurs in the environment outside of the hosts, however NT within the squid light organ has yet to be demonstrated (Pollack-Berti et al., 2010). We here consider another mutualism involving vertical inheritance of symbionts in the lumbricid earthworm *Eisenia fetida* that, similar to the squid symbiont, provides opportunity for exchange outside the immediate symbiotically associated population.

*E. fetida* harbors strains of *Verminephrobacter eiseniae*, a Microbactericeae member, and a novel Cytophagales within their excretory nephridia (Davidson et al., 2010). This mixed bacterial consortium is transferred during reproduction into the egg capsule where these cells colonize the developing earthworms (Davidson and Stahl, 2008). The egg capsule contains earthworm embryos and a dense (∼10^9^ cells per mL) microbial population with both known symbiotic bacterial species and possible contaminants from the soil (Davidson et al., 2010). The composition of the community in the albumin changes over the course of development from dominance by *Verminephrobacter* (∼70% of the population; Davidson and Stahl, 2008), to abundance of other cell types (i.e. *Herbaspirillum, Sphingomonas* and *Chitinophaga*; Davidson et al., 2010). Thus, within both the nephridia and the egg capsules, there is opportunity for genetic exchange among the microbial community members. The presence of foreign genes within the *Verminephrobacter* (VeEf1-2) genome, some of which have potential functions for *V. eiseniae* as a member of earthworm nephridial consortium (Pinel, 2009), provides evidence that gene exchanges occur.

*Verminephrobacter eiseniae* possesses type IV pili (TFP) that are required for successful colonization of the earthworm embryo (Dulla et al., 2012). The machinery employed to synthesize and retract pili is implicated in the uptake of DNA by naturally competent gram negative bacteria with TFP, Com type IV (involve assembly of pseudopili), and type II secretion systems (Palmen et al., 1993; Baur et al., 1996; Fussenegger et al., 1997; Hofreuter et al., 2000, 2001; Averhoff and Friedrich, 2003; Chen and Dubnau, 2004; Thomas and Nielsen, 2005; Johnsborg et al., 2007). The study presented here follows from earlier observations that *Acidovorax* species, close free-living relatives of *V. eiseniae,* showed evidence of natural competence in culture (Pinel, 2009). First, we tested *V. eiseniae* for the ability to incorporate free DNA under different culture conditions to evaluate influences of nutrients and density on competence. Then, mutants with defects of the TFP genes were evaluated to determine the possible role of the TFP in DNA uptake. Finally, the relevance of natural competence was tested within the host system by evaluating the uptake of free DNA by *V. eiseniae* within freshly deposited *E. fetida* egg capsules. Demonstration of DNA uptake within the host system would provide evidence for genetic mixing within the host environment of an obligate mutualist, which has implications for adaptive evolution of these ubiquitous symbionts of lumbricid earthworms.

## MATERIALS AND METHODS

### BACTERIAL STRAINS AND GROWTH CONDITIONS

All bacterial strains were cultured on a complex medium, *Acidovorax* Complete Medium (ACM; Pinel et al., 2008) solidified with 1.5% Phytagel at 28∘C (Pinel et al., 2008). For cultures of *Acidovorax* species *A. temperans* (ATCC 49665^T^), *A. facilis* (ATCC 11228^T^), *A. defluvii* (DSM 12644^T^), and *A. delafieldii* (ATCC 17505^T^), kanamycin and streptomycin were used at 125 and 150 μg/mL, respectively. For the spontaneously rifampicin resistant *V. eiseniae* EF05-2r, kanamycin, gentamycin and rifampicin were used at concentrations of 25 μg/mL, 15 μg/mL, and 100 μg/mL, respectively. *E. coli* Top10 (Invitrogen Life Technologies, USA) and S17-1 were routinely maintained on Luria Bertani (LB) medium at 37∘C with 50 μg/mL kanamycin.

### EARTHWORM CULTURE

Adult and juvenile *E. fetida* were maintained as previously described (Davidson and Stahl, 2006; Davidson et al., 2010). Egg capsules were collected and maintained on filter paper moistened with distilled water in Petri dishes at 21∘C.

### MUTANT CONSTRUCTION

Spontaneous streptomycin resistant *Acidovorax* mutants were obtained by plating overnight cultures of *A. facilis* or *A. temperans* on ACM streptomycin plates. One isolated resistant mutant culture was preserved for each species, and randomly mutagenized via insertion of the Tn5 Km^R^-DNA present on pRL27 (Larsen et al., 2002). Resulting Tn5 insertion mutants were harvested and pooled, and used as the source of donor DNA for NT assays.

Disruption of *pilT* (ORF 01909 EF01-2) of *V. eiseniae* EF05-2r was generated by insertion of the kanamycin resistance marker. DNA fragments, upstream and downstream of the pilT gene, ∼1 Kb in size were amplified from EF05-2r genomic DNA, using primers pilTsec1FwdGD (5′-GCC CTA GGT CGC TCG CTC CGG TAT GCG GCG C-3′), pilTsec1RevGD (5′-GCA AGC TTG GCG CGC TTT CGC GTC AAC GCC-3′), Xho1pilTFwd (5′-GCC TCG AGC CCT CCA TCG TTG CGG GTG A-3′) and Xba1pilTRev (5′-GCT CTA GAA AGG CCG CAT CAG CGT GCA G-3′). Fragments were cloned into pENTR/D-Topo-MCS:kan (Invitrogen Life Technologies, USA) flanking the kanamycin resistance gene to create pENTR/D-Topo-MCS:kan*pilT*. NT of *V. eiseniae* EF05-2r was performed, described below, and mutants were screened for marker insertion. A mini-Tn5 transposon conferring kanamycin resistance was introduced into *V. eiseniae* EF05-2r from *E. coli* S17-1 (pRL27; Larsen et al., 2002) using standard methods.

Natural transformation of *V. eiseniae* EF05-2r was performed, described below, and mutants were screened for marker insertion.

### DNA ISOLATION

Donor DNA for *Acidovorax* transformations was extracted via a modified Marmur protocol as described (Pinel et al., 2008). DNA was fragmented to a size range ∼2–20 Kb in a TE buffer (pH 8.0)/25% glycerol solution with a stream of N_2_ at 10 psi for 2 min in plastic nebulizers (Invitrogen, Carlsbad, CA, USA) or enzymatic fragmentation using Sau3AI. Following fragmentation, DNA was ethanol-precipitated and resuspended in TE buffer.

Donor DNA for *Verminephrobacter* transformations was isolated with the DNAeasy (Qiagen) extraction kit from a collection of random Tn5 insertion mutants. DNA was fragmented via partial digestion with Sau3AI. Fragments ranging from 2–5 Kb were gel purified and stored in Tris buffer pH 7.2. Donor DNA for *Verminephrobacter* transformations was also harvested from *E. coli* Top10 harboring the appropriate plasmid. A PCR Phusion master mix (New England Biolabs) was used for all PCR reactions.

### NATURAL TRANSFORMATION IN CULTURE

Cells were scraped from ACM plates after growing for 10 days, the time it takes *V. eiseniae* to form colonies, rinsed with mineral salts media (MSM; 25 mg NaCl, 5 mg KH_2_PO_4_, 1 mg MgSO_4_, 0.1 mg CaCl_2_ per liter, pH 7.2–7.4), resuspended with appropriate media and incubated at 28∘C overnight. Cultures were adjusted to an OD_600_ of 1.0 for all conditions. Triplicate samples were used for each replicate treatment, replicates were prepared from separate cultures. *Acidovorax* or *V. eiseniae* EF05-2r genomic DNA from Tn5 mutants was added at 10 μg/mL to suspended cells. DNA-free controls were included in all experiments. Samples were incubated at 28∘C for 30 min unless noted otherwise. Uptake of free DNA was halted by addition of 2 U/mL DNaseI. Following incubation and DNaseI addition, serial dilutions were spot-plated (20 μl/spot; 4 replicates/dilution) on ACM with appropriate antibiotics. A non-selective ACM plate was included to confirm viable cell density.

#### Selection of experimental NT conditions

Natural transformation in *V. eiseniae* EF05-2r was evaluated using pENTR/D:MCSkan-*pilBC* to determine optimal DNA concentrations, incubation times, and the influence of cell density and nutrients on natural compentence rates. All reactions were plated on non-selective and selective media to test for cell viability and transformation frequency, respectively. DNA (0.033 ng/μl, 0.83 ng/μl, 1.67 ng/μl, and 3.33 ng/μl in 30 μl) was mixed with *V. eiseniae* EF05-2r cell solution (OD_600_ 1.0) and transformants were recovered at multiple time points between 0 and 24 h, only data from 6 to 24 h are shown.

#### Influence of nutrients and cell density on NT

Cultures of *Acidovorax* spp. tested for transformation were grown in ACM broth shaking at 250 rpm (Pinel et al., 2008) to OD_600_ ∼1.0 at 28∘C. When appropriate, kanamycin and streptomycin were used at 125 and 150 μg/ml respectively. For tests in nutrient-limited medium, cells were collected via centrifugation, rinsed and resuspended in equal volumes (unless noted otherwise) of minimal medium (20 mM MOPS buffer pH 7.2; per liter 1.0 g NaCl, 0.1 g MgSO4, 0.1 g CaCl2, 1 ml Trace Minerals Solution SL12). For competence assays, 0.5–1.0 ml cell suspension aliquots in 13 mm × 100 mm glass tubes (VWR) in triplicate were used for each replicate (separate cultures). Triplicate samples were used for every treatment. If present, DNA was added at 10 μg/mL. DNA-free and DNaseI-treated controls were included in all experiments except on time series. Samples were incubated for 30 min unless noted otherwise. Uptake of free DNA was halted by addition of 2 U/mL DNase I. Following incubation and DNase I addition, serial dilutions were spot-plated (20 μl/spot; 4 replicates/dilution) on ACM-1.5% Phytagel plates. Colonies were counted under a dissecting microscope after 30–40 h of incubation.

To assess nutritional effects on transformation frequencies in *V. eiseniae*, strain EF05-2r cells were suspended in MSM supplemented with either 5 mM KH_2_PO_4,_ 20 mM NH_4_Cl, D-mannose, galactose, fructose, L-fucose, D-arabinose, hydroxy butyric acid (HBA), or pyruvate. Mixtures were incubated overnight at 28∘C. pENTR/D:MCSkan-*pilBC* was then added to a final concentration of 0.667 ng/μl and transformants were recovered after 24 h of incubation.

The influence of cell density on transformation rates in *V. eiseniae* EF05-2r was evaluated by serial dilution of cells to 10^9^, 10^8^, 10^7^, 10^6^, and 10^5^ cells/mL in ACM and incubation overnight at 28∘C. The following day, DNA was added to a final concentration of 0.667 ng/μl and transformants were recovered at 6 and 24 h, and plated as described above.

### INFLUENCE OF HETEROLOGOUS DNA ON TRANSFORMATION

*Acidovorax* spp. were tested for specificity of DNA for NT substrate by first establishing streptomycin spontaneous resistant mutants obtained after plating overnight cultures of *A. facilis* or *A. temperans* in ACM-Phytagel-Streptomycin plates. Resulting colonies were streaked for isolation. One isolated resistant mutant culture was preserved for each species, and used as a recipient of DNA from Tn5-transformed Km^R^-DNA donor cells in assays for DNA NT assays. If appropriate, DNA and DNAaseI were added as above. Incubations for DNA uptake/transfer were extended to 1 h. Reactions were terminated, and cell suspensions plated as above in plates with streptomycin and with/without kanamycin to test for acquisition of resistance from donor DNA.

Specificity of DNA uptake by *V. eiseniae* was tested indirectly by adding heterologous DNA in increasing amounts to bind non-specific DNA binding sites. Inhibition of transformation by *V. eiseniae* DNA in the presence of heterologous DNA was tested by adding increasing amounts of sheared salmon sperm DNA (Ambion; 20 ng, 200 ng, 2 μg, 20 μg, and 30 μg per 30 μl) to a NT reaction containing 20 ng pENTR/D:MCSkan-*pilBC* per 30 μl. Reactions were incubated for 24 h and transformants were recovered as described above.

### NATURAL TRANSFORMATION IN EGG CAPSULES

Egg capsules (0–3 days old) were harvested from earthworm colonies wherein *V. eiseniae* EF05-2r is the sole *Verminephrobacter* strain colonizing the nephridia and egg capsule (Dulla et al., 2012). Capsules were rinsed with water to remove adhered particulates from bedding. Capsules were air dried until a small dimple appeared on the surface of the egg indicating water loss. Approximately 1–3 μl of a 100 ng/μl pENTRD:MCSkan-*pilBC* solution was injected into the capsule with a 30 G needle until re-inflated. Eggs were again air dried to reseal the puncture left by the needle. Capsules were incubated at 25∘C for 24 h and then macerated in MSM. DNAseI was added to a concentration of 2 U/mL and incubated for an hour at room temperature. Serial dilutions of contents were then plated on ACM plates; rifampicin (100 μg/mL) and benlate (10 μg/ml; prevents fungal growth) to select for *V. eiseniae* EF05-2r and additional kanamycin 25 μg/mL for selection of transformants. Reactions containing less than 10^3^ CFU/egg were not included in analysis.

### ELECTRON MICROSCOPY

Cells were grown on ACM plates, collected and suspended in 1.25% glutaraldehyde, 0.1 M sodium phosphate buffer, pH 7.3, overnight at 4∘C, washed in buffer, and post fixed with 2% osmium tetroxide, in 0.05 M phosphate buffer 1.5 h, rinsed and stored in 0.1 M phosphate buffer, pH 7.3. Samples for TEM (transmission electron microscope) were mounted on 150 mesh rhodium/copper grids, stained with uranyl acetate and lead citrate and examined using a JEM 1200EX II TEM (JEOL Ltd, Tokyo, Japan).

### STATISTICAL ANALYSIS

General statistics and one-way ANOVA using JMP 8 (SAS Institute, Carey, NC, USA) was used to determine effects of treatments on NT frequencies.

## RESULTS

### NATURAL TRANSFORMATION IN COMPLETE MEDIA AND MINERAL SALTS SOLUTION

*Verminephrobacter eiseniae* and *Acidovorax* strains (listed in **Table 1**) were incubated with exogenous DNA derived from a library of mutants containing random genomic insertions of a kanamycin resistance gene introduced using the hyperactive Tn5 system encoded by the pRL27 plasmid (Larsen et al., 2002). Kanamycin resistant colonies of *V. eiseniae* EF05-2r and *A. temperans* appeared on ACM (**Table 1**), indicating their ability to take in and incorporate the free DNA. Incorporation was confirmed by PCR amplification of an internal fragment of the mini-Tn5 construct. Cells incubated without added exogenous DNA showed no spontaneous resistant mutants arising from kanamycin selection. None of the tested *Acidovorax* strains (*V. eiseniae* was not tested) showed detectable transformation when incubated with DNA from a different *Acidovorax* species.

**Table 1:** Natural transformation frequency^a^.

Strain	ACM	MSM
*Acidovorax temperans*	1.3 × 10^-7^ ± 9.0 × 10^-8^	2.5 × 10^-5^ ± 2.6 × 10^-6^
*A. facilis*	n.d.	7.1 × 10^-6^ ± 3.0 × 10^-6^
*A. defluvii*	n.d.	n.d.
*A. delafieldii*	n.d.	n.d.
*Verminephrobacter eiseniae* EF05-2r	8.8 × 10^-7^ ± 1.0 × 10^-6^	1.9 × 10^-6^ ± 1.1 × 10^-6^

a Bacteria were grown in nutrient rich (ACM) or deficient (MSM) media prior to incubation with 10 μg/mL sheared genomic DNA from Tn5 mutants for 30 min, n.d., none detected, *n* ≥ 4.

In Mineral Salts Medium (MSM) lacking carbon and nitrogen, transformants of *A. facilis* appeared, and the frequencies of NT for *A. temperans* and *V. eiseniae* EF05-2r increased. Notably, the transformation frequency of *A. temperans* increased about 200-fold in the MSM medium relative to that observed for ACM (**Table 1**) and was shown to increase with time following transfer from ACM to MSM (Supplementary Figure 1A). The highest transformation frequency was transient and observed at 325 min, after which the frequency drops.

### INFLUENCE OF CARBON SOURCES ON NATURAL TRANSFORMATION OF *V. EISENIAE* EF05-2R

The influence of nutrient status on the transformation of *V. eiseniae* EF05-2r was evaluated using a plasmid construct (pENTR/D-Topo-MCS:kan*pilBC* ) containing 1kb of this organism’s *pilB* and *pilC region* and a neomycin phosphotransferase gene (*npt2)* inserted between *pilB* and *pil*C. Integration of *pilB:npt2:pilC* into the *V. eiseniae* TFP gene cluster via a double crossover was confirmed in a subset of transformants by PCR amplification of the pUC origin (origin of replication for the plasmid used). The absence of amplifiable vector in the tested transformants was consistent with double crossover integration.

After testing several DNA concentrations (1, 25, 50, 100 ng/uL) and two incubation times (6 and 24 h) for transformation frequency of *V. eiseniae* EF05-2r in ACM (**Table 2**), a single DNA concentration (0.66 ng/uL, ∼100 copies per cell) and incubation period (24 h) were selected to evaluate the influence of carbon source on transformation. Under these conditions, transformation by pENTR/D-Topo-MCS:kan*pilBC* in MSM resulted in a two-fold increase in frequency compared to ACM (**Table 3**). Transformation frequency in MSM supplemented with 20 mM NH_4_Cl was not significantly different than ACM (**Table 3**). All carbon sources tested (**Table 3**) were previously shown to support growth of *V. eiseniae* [9]. After 24 h incubation with any one of the carbon sources except galactose, transformation frequencies decreased by at least two-fold (**Table 3**). The inclusion of 20 mM pyruvate in MSM resulted in a 24-fold decrease in frequency.

**Table 2:** Transformation frequency of *V. eiseniae* 05-2r with increasing donor DNA.

DNA (ng) added^a^	6 h^b^	24 h^b^
1	9.4 × 10^-6^ ± 1.4 × 10^-5^ (A)	5.9 × 10^-5^ ± 3.8 × 10^-5^ (B)
25	8.6 × 10^-5^ ± 7.3 × 10^-5^ (B)	3.8 × 10^-4^ ± 1.9 × 10^-4^ (C)
50	1.6 × 10^-4^ ± 8.5 × 10^-5^ (C)	1.3 × 10^-3^ ± 9.4 × 10^-4^ (D)
100	2.0 × 10^-4^ ± 1.6 × 10^-4^ (C)	1.3 × 10^-3^ ± 1.2 × 10^-4^ (D)

a pENTR/D-Topo-MCS:kan*pilBC* added to 30 μl reaction incubated at 25∘C.

b Mean transformation frequency in ACM ± SD, *n* = 8. Values with different letters differ significantly (*p* < 0.05).

**Table 3:** Nutrient effects on transformation frequencies of *V. eiseniae* 05-2r.

Supplemented nutrient^a^	Trial 1^b^	Trial 2^b^
MSM	8.6 × 10^-4^ ± 2.0 × 10^-4^ (X)	6.6 × 10^-4^ ± 2.2 × 10^-5^ (A)
ACM	4.2 × 10^-4^ ± 1.2 × 10^-4^ (Y)	
NH_4_Cl	9.3 × 10^-4^ ± 1.1 × 10^-4^ (X)	
D-mannose	4.8 × 10^-4^ ± 1.1 × 10^-4^ (Y)	3.2 × 10^-4^ ± 5.6 × 10^-5^ (B)
Galactose		6.1 × 10^-4^ ± 7.7 × 10^-5^ (A)
Fructose		2.2 × 10^-4^ ± 1.4 × 10^-4^ (BC)
L-fucose		2.2 × 10^-4^ ± 2.6 × 10^-5^ (BC)
D-arabinose		1.4 × 10^-4^ ± 1.1 × 10^-4^ (CD)
β-Hydroxybutyrate		9.1 × 10^-5^ ± 9.0 × 10^-5^ (D)
Pyruvate		2.7 × 10^-5^ ± 9.0 × 10^-6^ (D)

a Nutrients added to a concentration of 20 mM. 20 ng pENTR/D-Topo-MCS:kan*pilBC* added to 30 μl reaction incubated at 25∘C for 24 h. Cell density adjusted to OD_600_ of 1.

b Mean transformation frequency ± SD, *n* = 4. Values with different letters differ significantly (*p* < 0.05).

### INFLUENCE OF CELL DENSITY ON NATURAL TRANSFORMATION OF *V. EISENIAE* EF05-2R

**FIGURE 1:**
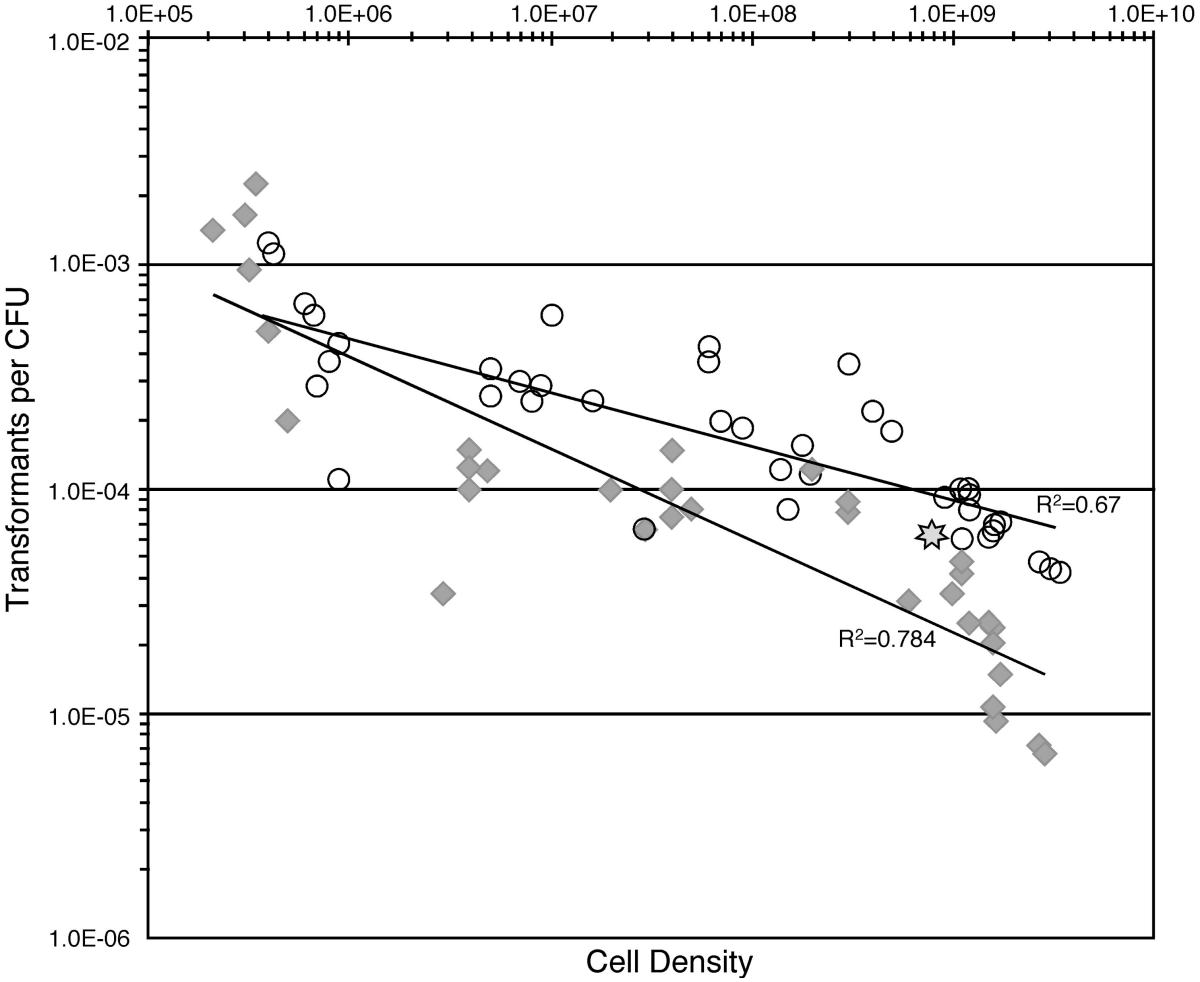
**Relationship of cell-density and natural transformation rates when incubated in a complete medium ACM.** Open circles, results using 24 h incubations; gray diamonds, results using 6 h incubations. Star, density and transformation rate determined in the *E. fetida* egg capsules.

*Verminephrobacter eiseniae* EF05-2r cells harvested from ACM plates were resuspended at increasing densities and allowed to grow for 24 h in ACM before being tested for natural competence. Testing these cells should represent the influence of density on natural competence. Incubation of these cells with DNA for either 6 hr or 24 h showed differences at the highest cell densities, with 6 hr yielding fewer transformations. Transformation frequencies were sensitive to cell densities and decreased 30-fold when cell density is increased from 4 × 10^5^ to 3 × 10^9^ cells/mL for a 24 h incubation, and approximately 200-fold when incubated for only 6 h (**Figure 1**).

### INFLUENCE OF TFP MUTATIONS ON NATURAL TRANSFORMATION

**FIGURE 2:**
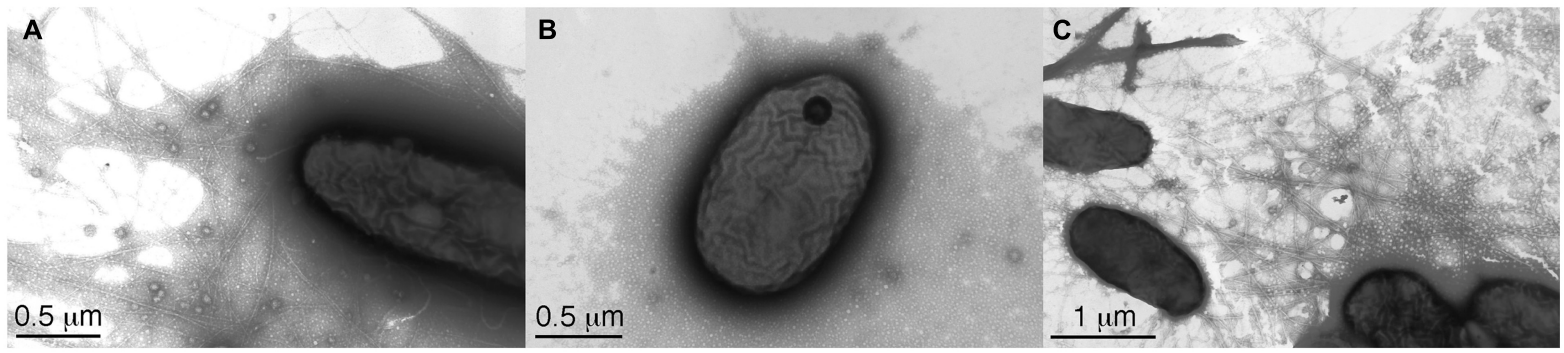
**Transmission election micrographs of **(A)**, *Verminephrobacter eiseniae* EF05-2r displaying pili, **(B)**, *V. eiseniae* EF05-2r *pilBC-* lacking pili, and **(C)**, *V. eiseniae* EF05-2r *pilT-,* with abundant pili**.

Two *V. eiseniae* EF05-2r mutants with interrupted *pilB, pilC,* and *pilT* genes (all components of the TFP machinery) were tested for their ability to take up DNA. A traffic NTPase involved in assembly of the type IV pili is coded by *pilB*, and an inner membrane platform protein critical for the export and assembly of the pili components is coded by *pilC* (Takhar et al., 2013). *PilT* codes for a traffic ATPase involved in disassembly for retraction of the pilus (Watson et al., 1996). The mutant *V. eiseniae* EF05-2r*pilBC-* (*pilB* and *pilC* coding regions truncated) lacks visible pili by TEM and no longer displays twitching motility (Dulla et al., 2012). Interruption of *pilT* resulted in no visible twitching movement in twitching assays (data not shown), even though abundant production of pili was observed by TEM as large aggregates with masses of pili indicating hyper-piliated cells (**Figure 2**). This phenotype is consistent with the described function of PilT, with its deletion resulting in loss of pilus retraction (by disassembly) and hyperpiliation (Nunn et al., 1990; Strom and Lory, 1993).

Incubation of WT *V. eiseniae* EF05-2r non-piliated *V. eiseniae* EF05-2r*BC*-, and hyper-piliated EF05-2r*pilT-* cells with a *ptsI*s1:gent:*pstI*s2 construct (pENTR/D-Topo-MCS:gent*ptsI*) conferring gentamicin resistance, resulted in NT frequencies of the WT comparable to prior experimentally determined levels, but a significant decrease in NT frequencies for each of the TFP mutants (**Table 4**). Expressing an antibiotic resistance gene, such as *npt2*, can add metabolic load on bacterial metabolism. To control for the possible influence of kanamycin resistance gene *npt2* on mutant performance, *V. eiseniae* EF05-2r*flgKL-* (lacking functional flagella) was tested for transformation frequency and resulted in a twofold reduction in NT rates relative to wild type but remained significantly higher than for the TFP mutants.

**Table 4:** Natural transformation of TFP mutants.

Strain	TFP^a^	Transformation frequency^b^
EF05-2r	+	1.2 × 10^-2^ ± 5.2 × 10^-3^ (A)
VE*pilBC*-	^-^	1.3 × 10^-7^ ± 7.3 × 10^-8^ (C)
VE*pilT*-	++	3.2 × 10^-8^ ± 3.2 × 10^-8^ (C)
VE*flgKL-*	+	5.2 × 10^-3^ ± 1.1 × 10^-3^ (B)

a +, Presence of TFP; ++, hyper-piliated; -, no TFP; presence of TFP confirmed by TEM.

b Mean transformation frequency ± SD, *n* = 4. Strains tested with 20 ng pENTR/D-Topo-MCS:gent*ptsI*/ 30 μl MSM. Values with different letters differ significantly (*p* < 0.05).

### DNA SEQUENCE SPECIFICITY AND INTERFERENCE BY NON-HOMOLOGOUS DNA

Some species of bacteria have the ability to select homologous DNA for uptake, one of a number of mechanisms theorized to protect bacteria from excessive horizontal gene transfer (Thomas and Nielsen, 2005). Natural transformants of *A. temperans* were only observed when donor DNA of the same species was used. Addition of marker DNA from *A. facilis, A. defluvii,* or *A. delafieldii* did not result in Km^R^ CFU frequency above background (not shown).

**FIGURE 3:**
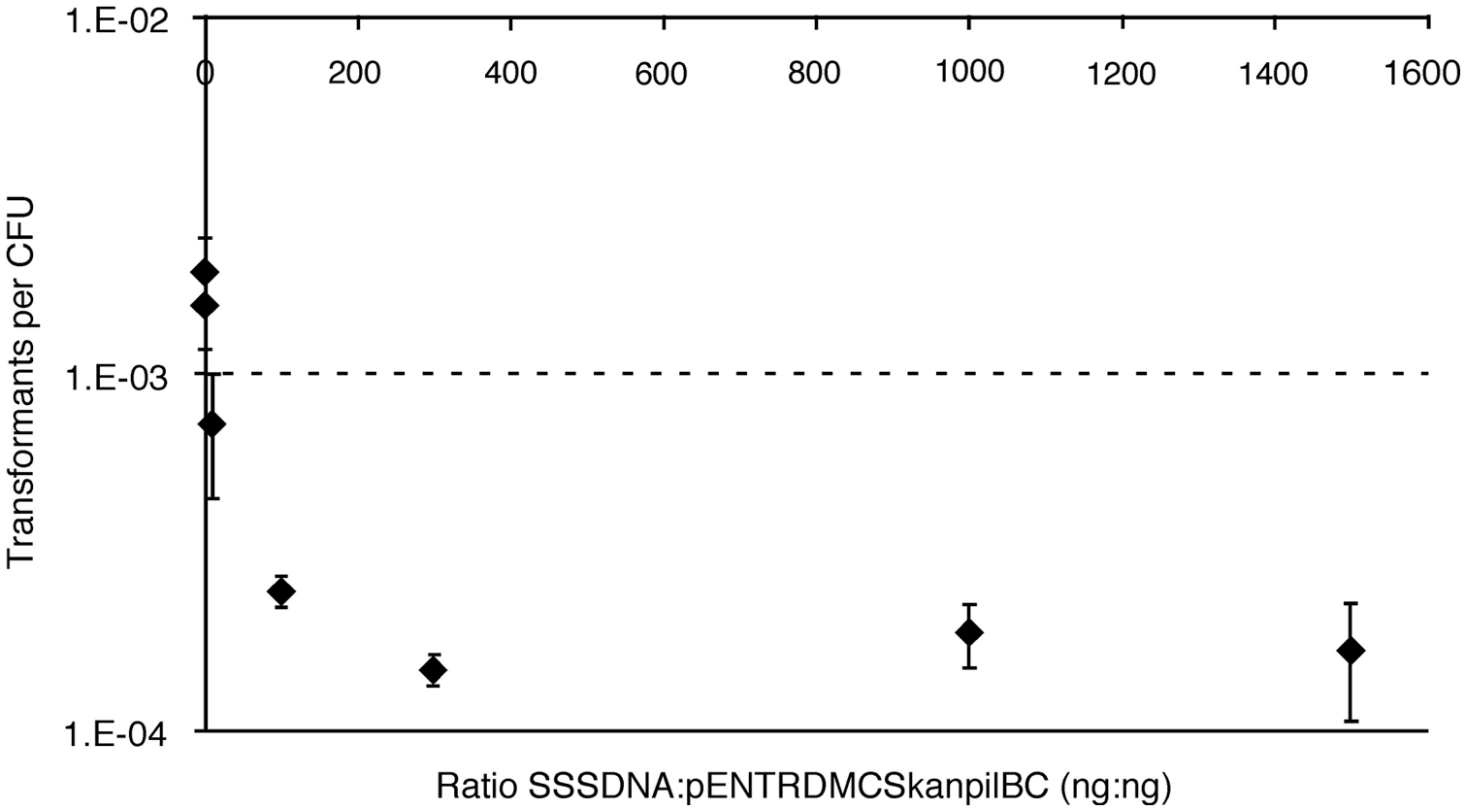
**Natural transformation rates in the presence of increasing concentrations of heterologous DNA.** Sheared salmon sperm (SSS) DNA was added in increasing amounts to NT reactions containing 20 ng pENTR/D-Topo-MCS:kan*pilBC.* Each point is an average of *n* = 4 samples, error bars represent one SD.

To evaluate whether DNA binding and uptake by *Verminephrobacter* is promiscuous or specific, cells were incubated with increasing amounts of unrelated DNA (sheared salmon sperm DNA, SSSDNA) in the presence of the homologous gene construct pENTR/D-Topo-MCS:kan*pilBC*, and evaluated for transformation. The hypothesis is that the presence of abundant DNA would bind the DNA binding sites and interfere with uptake of the DNA marker if binding for uptake is non-specific. DNA uptake due to binding of specific signal sequences would result in only limited reduction in NT. Addition of 20 ng SSSDNA, a 1:1 ratio SSSDNA:pENTR/D-Topo-MCS:kan*pilBC*, resulted in an insignificant decrease in transformants, with the maximum effect of about 10-fold decrease at a 100:1 ratio. This lower frequency of transformation did not change with increasing SSSDNA concentration, up to the maximum ratio tested of 30 μg:20 ng (1500:1; **Figure 3**). These data suggest there is selective DNA binding prior to uptake.

### NATURAL TRANSFORMATION IN EGG CAPSULES

Capsules from an earthworm colony colonized by a single *V. eiseniae* strain, EF05-2r (Rifampicin resistant) were used to demonstrate NT in earthworm egg capsules. Capsules (0–1 day old), each containing approximately 5 μl of albumin, have an estimated 9.0 × 10^5^ bacterial cells per μl of albumin, with approximately 3.2 × 10^6^ *V. eiseniae* cells per capsule or 6.4 × 10^8^ cells/mL (Davidson and Stahl, 2008). Approximately 100 ng pENTR/D-Topo-MCS:kan*pilBC* was injected into capsules and incubated for 24 h prior to recovery. Resultant transformants were verified with PCR. The estimated transformation frequency was 4.5 × 10^-5^± 4.9 × 10^-5^ (*n* = 8). This rate is similar to those observed in culture at a similar cell density of 7 × 10^8^ (**Figure 1**). Control experiments without injected DNA resulted in no detectable transformants (*n* = 4).

## DISCUSSION

Natural transformation has been well studied in several pathogens to elucidate mechanisms of DNA uptake (Catlin, 1960; Hofreuter et al., 2000; Chen and Dubnau, 2004; Johnsborg et al., 2007; Frye et al., 2013), but demonstrated for only one beneficial symbiont in culture, and not yet within the host (Pollack-Berti et al., 2010). In the study presented here, we demonstrate that a symbiotic member of an earthworm nephridial consortium is able to import and incorporate free DNA into the genome while within the host environment, that type IV pili mediated specific DNA uptake, and that DNA uptake is regulated by nutrients and cell density. We also showed that some members of *Acidovorax*, the closest free-living relatives of *Verminephrobacter*, are also naturally competent. This ability would enable *V. eiseniae* to access DNA from other organisms, providing a mechanism for genetic innovation. DNA released from diverse microorganisms present in the egg capsules – including the three nephridial symbionts of *E. fetida* and soil bacteria introduced into the egg capsules – provides a rich environment for DNA exchange.

For bacterial cells, the process of DNA binding and guidance into the cytoplasm can be mediated by surface and transmembrane protein components of the type II, type IV (TFP) and the Com type IV secretion systems (Wolfgang et al., 1998; Averhoff and Friedrich, 2003; Chen and Dubnau, 2004; Johnsborg et al., 2007; Cehovin et al., 2013). The dramatic reduction in transformants after disruption of *V. eiseniae* TFP genes clearly implicates the pili apparatus in DNA import. Hyperpiliated *pilT* mutants of *Pseudomonas stutzeri* were still able to bind DNA non-specifically yet unable to uptake DNA (Graupner and Wackernagel, 2001), indicating both binding and retraction of pili are required for DNA uptake since PilT is involved in pilus retraction. However, binding and retraction of a pseudo pilus, rather than a full pilus, is thought to initiate import of the dsDNA (Chen and Dubnau, 2004). The loss of competency from the *V. eiseniae pilT* mutant was likely caused by reduction of pseudopili because synthesis of full pili is favored over pseudopili in *pilT* mutants (Averhoff and Friedrich, 2003). Without the *pilB* or *pilC*, neither structure can be synthesized, and the failure of *pilBC*- mutant to incorporate DNA at the wildtype levels confirms the role of the TFP in DNA uptake for *V. eiseniae* 05-2.

The development of natural competence is influenced by specific environmental factors, including nutrient availability, presence of specific substrates, cell density, and growth stage (Cohan et al., 1991; Lorenz and Wackernagel, 1994; Macfadyen et al., 2001; Meibom et al., 2005; Thomas and Nielsen, 2005; Johnsborg et al., 2007; Gulig et al., 2009; Pollack-Berti et al., 2010). Nutrients influence competence for *A. temperans* and *V. eiseniae*, demonstrated by increased rates of NT under low carbon source conditions and a significant decrease in transformation rates with addition of nutrients. The strength of the responses to specific nutrients varied, with pyruvate having a particularly strong suppression in *V. eiseniae* relative to other nutrients tested. The significance of the varied responses remains to be investigated. Density-dependent gene signaling also regulates induction of NT in several bacterial species, with examples of induction at either increasing or decreasing cell density (Havarstein et al., 1995; Wilson et al., 2003; Meibom et al., 2005). *V. eiseniae* rates of NT at high cell densities in culture were similar to rates in the egg capsule, also at a high density (10^9^ cells/ml; **Figure 1**), suggesting the cell density effect is consistent in both culture and the bacterium’s native environment.

The influence of nutrients and density on the competence of *V. eiseniae* shows potential for changes in DNA uptake by *V. eiseniae* throughout stages of the host life cycle. From the current data it is difficult to predict how DNA uptake rates would change considering the complexity of the capsule environment with shifting cells densities, nutrients and signals from the embryo and other microbes. Even with these variables, indications from culture tests are that the NT rate ranges between ∼10^-3^ and 10^-5^ cells per CFU, from nutrient replete to nutrient limited and low and high densities. The egg capsule NT rates in this study were measured in new, early development capsules with very small embryos, and the observed rate of 6.5 × 10^-5^ cell per CFU may vary as the environment changes during embryo development. The egg capsule contains a microbial community, composed of both the nephridial symbionts and members from the soil, whose composition changes over the course of development, likely altering microbial interactions and chemistry. The nutrient pool and chemistry change as the embryo consumes the albumin, matures and begins to excrete waste from the nephridia. The chemistry of this entire system from the start to end of development is currently undefined. The symbiotic cells are exposed to the mixed community in the albumin, but then shift to a simpler community within the nephridia of the host as part of a layered community in contact with host tissue. This transition from ampulla to egg capsule and into embryo is expected to influence the development of competence and therefore opportunities for uptake of DNA from different sources. The influence of the embryo developmental stage, and symbiont location, on the rates of NT remains to be investigated.

The occurrence of NT within the capsule provides evidence that DNA exchange among egg capsule community members is possible. However, our observation that transformation frequencies with homologous DNA was only slightly reduced in the presence of excess heterologous DNA provides evidence for selective DNA uptake in *V. eiseniae*. The pattern of NT inhibition by heterologous DNA was consistent with saturation of non-specific DNA binding regions on TFP, leaving specific sites available for binding of *V. eiseniae* DNA. If DNA binding was entirely non-specific, there would have been continued proportional decrease in NT resulting in a low NT rate, but this did not occur, and saturation happened at fairly low concentrations of SSSDNA. This finding suggests capsule community members contributing DNA to *V. eiseniae* may be limited by DNA sequence specificity. Selectivity of DNA uptake based on sequence is a well-established mechanism in Neisseriaceae and members of the Pasteurellaceae which limit DNA uptake using short (9–11 bp) DNA uptake sequences (DUS) recognized by TFP binding sites (Frye et al., 2013). This is considered a protective mechanism for prevention of genome damage caused by incorporation of random free DNA. Although incorporation of new DNA into the chromosome can be adaptive, it often leads to deleterious mutations. Thus bacteria have mechanisms to control the incorporation of exogenous DNA by selective binding, and regulation of uptake, degradation and/or incorporation (Thomas and Nielsen, 2005; Treangen et al., 2008).

DNA uptake sequence (DUS) mediating exchange of DNA among members of the same species has been postulated to enable *Neisseria gonorrhoeae* and *H. influenza* to stay ahead of the immune system by altering surface features to increase antigenic variability (Hobbs et al., 1994; Fudyk et al., 1999; Hamilton and Dillard, 2006). However, the genomes of *Neisseria* species also contain foreign genes, some contributing to virulence. Analysis of the location of foreign genes relative to known DUS sequences revealed that the conserved core genes were associated with the DUS, but not the foreign genes. These DUS sequences appear to mediate incorporation of genes with essential functions, the disruption of which during recombination events would be deleterious, thus maintaining genome stability rather than adaptive variation (Treangen et al., 2008). Thus species-specific DNA uptake is hypothesized to be a mechanism to maintain the integrity of core genome function in genomes that frequently recombine, rather than a mechanism for rapid innovation through acquisition of foreign genes.

The characteristics of the *V. eiseniae* EF01-2 genome are consistent with these bacteria having genome rearrangements, foreign gene uptake, and mechanisms that preserve core genome functions. *Verminephrobacter* species genomes examined to date show dramatic rearrangements that have eliminated synteny with closely related genomes and display accelerated rates of evolution (based on the 16S rRNA and rpoB genes) relative to free-living relatives (Pinel, 2009; Kjeldsen et al., 2012). Their evolution rates are comparable to other ancient vertically transmitted symbionts with similarly long host associations that show reduced genomes and increased AT bias (Lund et al., 2014). In contrast, the core genome of *Verminephrobacter* species have retained genome size, GC content and core functions, thus have changed more slowly than expected when compared to intracellular obligate symbionts (Lund et al., 2014). The ability to take up specific DNA from the environment would enable *V. eiseniae* to incorporate DNA to repair or replace genes damaged during frequent recombination events. Demonstration of NT for *V. eiseniae* with possible specific DNA exchanges within the host environment provides a mechanism for the genetic mixing model described in Lund et al. (2014), as a way to preserve the core genome as suggested by Treangen et al. (2008).

Genetic innovation and genome conservation are not mutually exclusive and horizontal gene transfer from outside a species does occur as shown by the presence of foreign genes in pathogens with DUS sequences. The same is likely true for *V. eiseniae*, and exchanges outside of the *V. eiseniae* population occur, albeit possibly rarely, and can provide innovative adaptations. For example, the *V. eiseniae* 01–2 genome contains genes matching those from other species including genes identified from *Xylella fastidiosa* (located on a plasmid) and seven distinct *pilY*-like adhesin genes, an unprecedented number, with high sequence similarities to *Xylella*, *Pseuodmonas,* and *Neisseria* species (Pinel, 2009). These foreign genes identified in *V. eiseniae* may provide advantages in the context of host interactions. The diverse *pilY* genes code adhesins potentially involved in binding to the host and to other bacterial cells at different locations in the earthworm during stages of the lifecycle. Within the populations of *V. eiseniae*, there is potential for continued genetic innovations and changes. Among the Lumbricidae, most genera tested carry *Verminephrobacter* together with a few other bacteria in their nephridia, but several species within *Lumbricus* and *Aporrectodea* harbor only *Verminephrobacter spp*. as their nephridial symbiont (Lund et al., 2010; Davidson et al., 2013). In such species there remains opportunity for gene exchanges within the *Verminephrobacter* pool, as these nephridial populations of *V. eiseniae* are not clonal, and within the egg capsules there are likely bacteria introduced from the soil as has been shown for *E. fetida* (Pinel et al., 2008; Davidson et al., 2010). The sequence specificity of these exchanges, and impacts on the dynamics of symbiont genome evolution promise to be an important area of investigation.

## Conflict of Interest Statement

The authors declare that the research was conducted in the absence of any commercial or financial relationships that could be construed as a potential conflict of interest.
