# Supplementary material for: Earthworm symbiont Verminephrobacter eiseniae mediates natural transformation within host egg capsules using type IV pili
Source: Front Microbiol. 2014 Oct 29;5:546. doi: 10.3389/fmicb.2014.00546 (PMC4212676; doi:10.3389/fmicb.2014.00546)
Supplement: Supplementary file 1 [file Data_Sheet_1.PDF]

Supplemental Materials

Earthworm symbiont *Verminephrobacter eiseniae* mediates natural transformation within the host egg capsules using type IV pili

Seana K. Davidson[1]*, Glenn F. Dulla[1], Ruth A. Go[1], David A. Stahl[1] and Nicolás Pinel[2]

[1]Department of Civil and Environmental Engineering, University of Washington, Seattle, WA 98195-2700, USA.

[2]Institute for Systems Biology, 401 Terry Ave N, Seattle, WA, 98109.

*Corresponding author

Additional data from experiments on Acidovorax species competency in response to nutrients

Cultures of *Acidovorax* spp. tested for transformation were grown with 250 rpm in ACM broth (Pinel et al., 2008) to OD_{600} ~1.0 at 28˚C. When appropriate, kanamycin and streptomycin were used at 125 and 150 µg/ml respectively. Cells were collected via centrifugation, rinsed and resuspended in equal volumes (unless noted otherwise) of nutrient-limited medium (20 mM MOPS buffer pH 7.2; per liter 1.0 g NaCl, 0.1 g MgSO4, 0.1 g CaCl2, 1 ml Trace Minerals Solution SL12) ammended when appropriate. Ammendments included (individually or jointly as indicated): sodium pyruvate (1 or 10 mM), casamino acids (1 g/L), NH4Cl (5 mM), and KH2PO4 (5 mM). For time series on nutrient-limited medium, resuspended cultures were incubated as per culture conditions.

Following transfer from ACM into MSM, *A. temperans* transformant frequencies increased over time (SFig. 1), with a peak in transformation rates at 325 minutes. This transient but reproducible increase suggests the competence is regulated by nutrient availability.

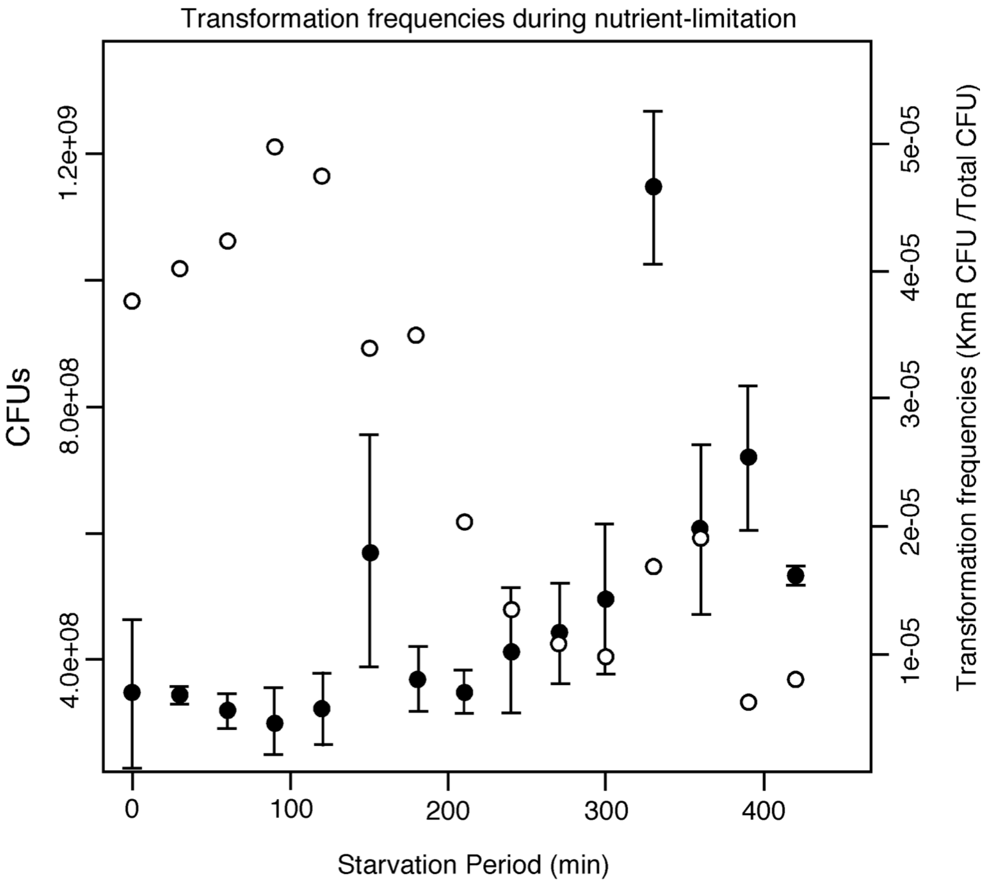

Supplemental Figure 1. Frequencies of naturally competent cells during nutrient-limited (starvation) conditions. The culture had been grown on ACM as described in the methods section. The time series represents intervals after resuspension in nutrient-limited medium. Filled circles, frequencies of natural transformation (Km^R CFU / total CFU); open circles, CFU. Error bars were omitted from CFU data points for clarity; average CFU standard deviation was $1.7x10^8$. The graph represents one of three experiments with similar results.

.

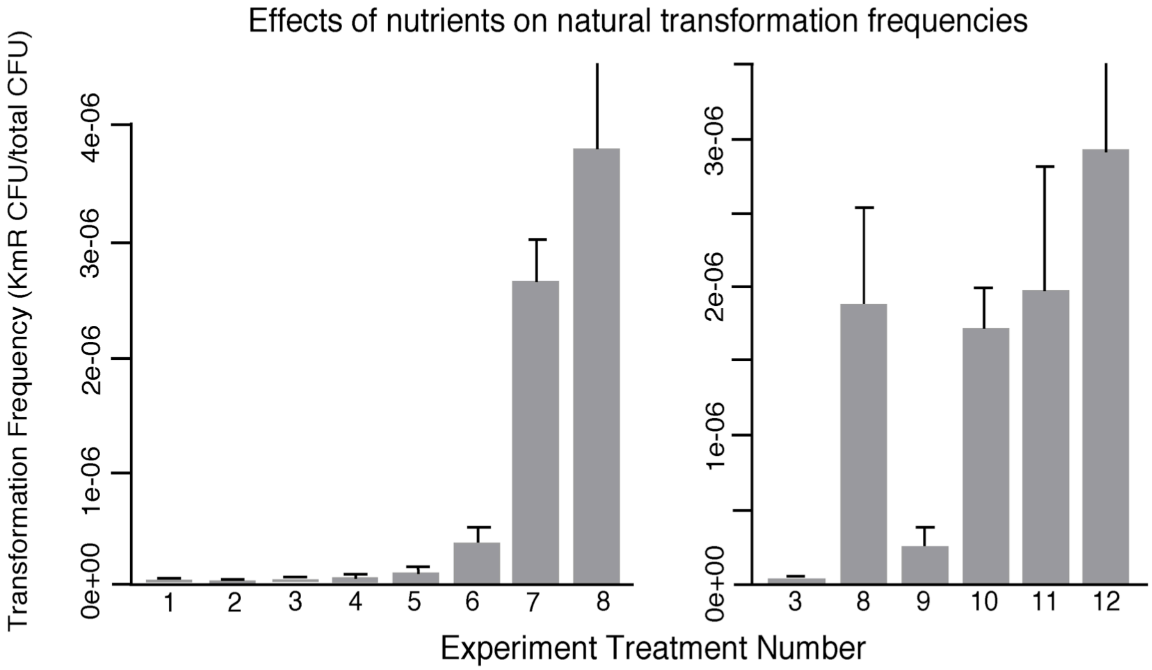

Supplemental Figure 2. Nutrient effects on natural competence of *Acidovorax temperans.* Treatment numbers correspond to: 1, Acidovorax complex médium (ACM); 2, reconstituted ACM (prepared from individual components sterilized separately); or minimal salts media ammended with: 3, sodium pyruvate/NH_4Cl/KH_2PO_4; 4, sodium pyruvate/NH_4Cl; 5, sodium pyruvate/KH_2PO_4; 6, NH_4Cl/KH_2PO_4; 7, sodium pyruvate (1 mM)/casamino acids (0.1 g/L); 8, no ammendments, 9, sodium pyruvate; 10, NH_4Cl; 11, KH_2PO_4; 12 casamino acids. Final concentrations are as indicated in the methods section, unless noted otherwise. Panel ‘a’ is one of two experiments; panel ‘b’ is a refinement of the previous two experiments. Because of the differences in absolute transformation frequencies, they are presented as separate panels.
